# More slices, less truth: effects of different test-set design strategies for magnetic resonance image classification

**DOI:** 10.3325/cmj.2022.63.370

**Published:** 2022-08

**Authors:** Mila Glavaški, Lazar Velicki

**Affiliations:** 1 Faculty of Medicine, University of Novi Sad, Novi Sad, Serbia; 2 Institute of Cardiovascular Diseases Vojvodina, Clinic for Cardiovascular Surgery, Novi Sad, Serbia

## Abstract

**Aim:**

To assess the effects of different test-set design strategies for magnetic resonance (MR) image classification using deep learning.

**Methods:**

Error rates in 10 experimental settings were assessed. The performance of pretrained models and data augmentation were examined as possible contributing factors.

**Results:**

Error rates in experimental settings using MR images of different patients for training and test sets were ten times higher than those in experimental settings using MR images of the same patients (four disease groups with whole-chest images, 46.80% vs 2.06%; four disease groups without whole-chest images, 49.09% vs 1.29%; sex classification with whole-chest images, 16.02% vs 0.96%; and sex classification without whole-chest images, 23.56% vs 0.30%). Error rates were higher when data augmentation was applied to settings that used MR images of different patients for training and test sets.

**Conclusion:**

When deep learning is applied to MR image classification, training and test sets should consist of MR images of different patients. Models built on training and test sets consisting of images of the same patients yield optimistic error rates and lead to wrong conclusions. MR images of neighboring slices are so similar that they cause data leakage effect.

Deep neural networks (DNNs), due to their ability to handle large amounts of complex data, are mostly used in the analysis of non-numerical data, such as image processing (1), including medical image analysis (2).

Machine learning should be applied critically and it is important to take into account all of its advantages and limitations (2,3). Deep learning is now entering the field of clinical diagnosis in order to provide a fully automated diagnosis (2). Despite many promising results of deep learning applications, multiple challenges hinder their use in clinical practice. One of them is the lack of evaluation methods for testing general performance in clinical settings (1).

Deep learning is a statistical method that learns only the statistics of the training data set. Once trained, the neural network can perform the learned mapping on unseen data (2). Performance evaluation tells us how well the model performs on unseen data (4). The test set is a set of data for model evaluation (5). DNNs perform very well on the training set and generalize suitably to a data set with identical or similar statistical distributions. However, the generalization ability can fail in unexpected ways if a data set with different statistical distributions is used for testing (for example, a different patient cohort). For clinical use, it is important that DNNs trained on one particular data set generalize well to other unseen data sets (2). It is also essential to train and evaluate the model on different representative data sets. Training and evaluation on the same data set introduces an optimistic bias, in which case we cannot be sure if the model just memorizes the training data or indeed generalizes well to unseen data (4).

Magnetic resonance imaging (MRI) (6) is widely used in clinical practice, and cardiac MRI is generally considered the reference standard method for evaluation of cardiac structure. Although MR images and medical images in general are rich sources of information, deep learning could empower them even further. Much of the current research focuses on deep learning applications in MR image analysis (2).

In contrast to the amount of research conducted in the field of deep learning application and performance in medical image analysis, far less attention has been paid to the design of test sets in such contexts. Medical image analysis using DNNs might represent a special class of computer vision problems because it introduces new challenges in image analysis. Furthermore, each error that affects diagnosis may harm patients. The aim of this study is to assess the effects of different test-set design strategies for MR image classification using deep learning.

## MATERIALS AND METHODS

### Data

We used Sunnybrook Cardiac Data (7) (https://www.cardiacatlas.org/studies/sunnybrook-cardiac-data/) consisting of cine-MR images of 45 patients from four disease groups. The disease groups were classified as follows (8):

1. Heart failure with infarction (HFI) group – ejection fraction <40% and evidence of late gadolinium enhancement (9 patients);

2. Heart failure without infarction (HF) group – ejection fraction <40% and no late gadolinium enhancement (12 patients);

3. Left ventricle hypertrophy (LVH) group – ejection fraction >55% and a ratio of ventricular mass over body surface area >83 g/m^2^ (12 patients);

4. Healthy (H) group – ejection fraction >55% and no hypertrophy (12 patients).

For each patient in the data set, sex and identification number were stated.

### The first set of experiments

Sunnybrook Cardiac Data MR images, originally in DICOM format, were converted to JPG format by a programmed DICOM to JPG converter, built using Pydicom and OpenCV-Python packages. Eight-bit depth was used.

**Figure 1:**
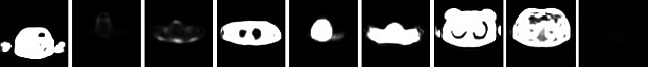
Examples of uninformative images excluded from further experiments.

Images ≤3 KB in JPG were excluded from further experiments because they were uninformative even for trained cardiologists or radiologists (examples are shown in Figure 1).

In all experiments, the FastAI library (9) was used. After image normalization (imagenet_norm) and data augmentation, ResNet-50 pretrained model (10) with one-cycle policy (11) was used (Table 1). Data augmentation consisted of a random flip, random rotation, random zoom, random lightning and contrast change, and random symmetric warp (Table 1).

**Table 1:** Data augmentation and model design

Option	Value
Library	FastAI
Image size	224
Batch size	20
Image normalization	imagenet_norm
Data augmentation	random flip flips limited to horizontal flips random rotation between -10 and 10 degrees with probability 0.75 random zoom between 1.0 and 1.1 with probability 0.75 random lightning and contrast change controlled by 0.2 with probability 0.75 random symmetric warp of magnitude between -0.2 and 0.2 with probability 0.75
Architecture	ResNet-50
Model fitting method	One cycle policy
Optimizer	Adam
Regularization	L2
Loss function	cross entropy loss

**Figure 2:**
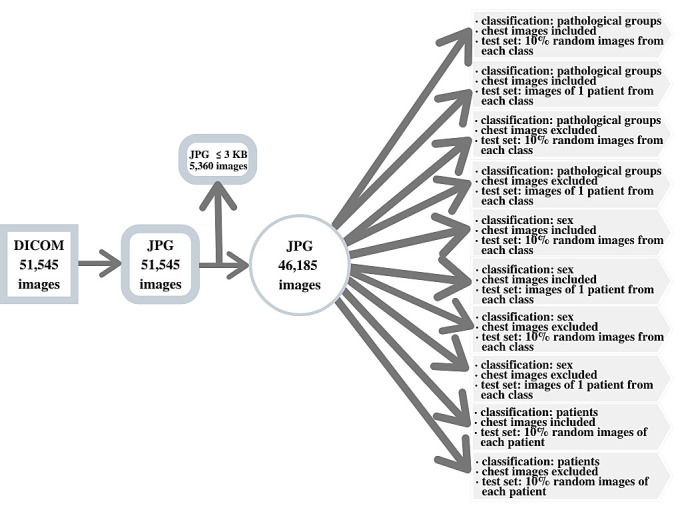
The first set of experiments.

In 10 experiments (Figure 2), three different classifications were performed, based on the following: 1) four disease groups (HFI, HF, LVH, and H), 2) sex (male and female), and 3) patients (each patient represented one class).

Since we estimated that the MR images showing the whole chest might mislead the models (and even a trained cardiologist or radiologist) in the classifications of heart diseases, we conducted all classifications twice: once including chest MR images and once excluding them from the training and test data sets. In the whole database, there were 708 chest images; chest images were provided for all patients.

Since the creation of a state-of-the-art-model was not a goal of this study, and since hyperparameter tuning for achieving better performance was not a part of our experiments, validation sets were not made.

Training and test sets were split manually using the holdout method. The effects of different test-set design strategies – using random images from each category and using images of different patients for training and test sets – were assessed by measuring the difference in error rates.

### The second set of experiments

**Figure 3:**
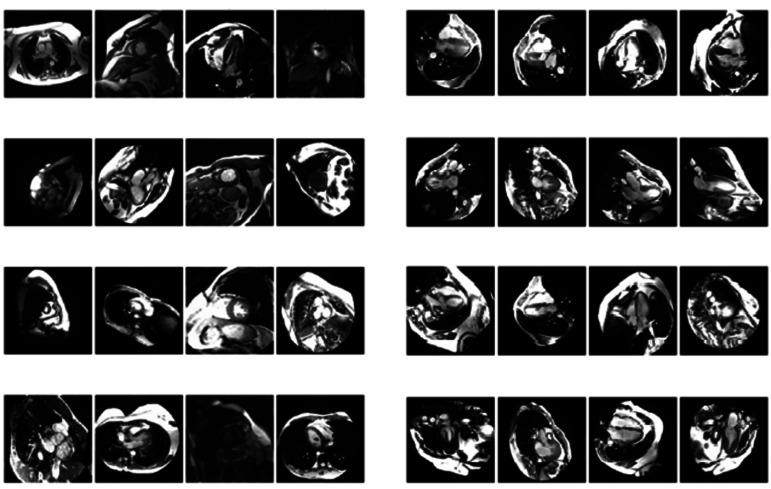
An example of images in one batch – when complete data set was used (left) and when uniform data subset was used (right).

**Figure 4:**
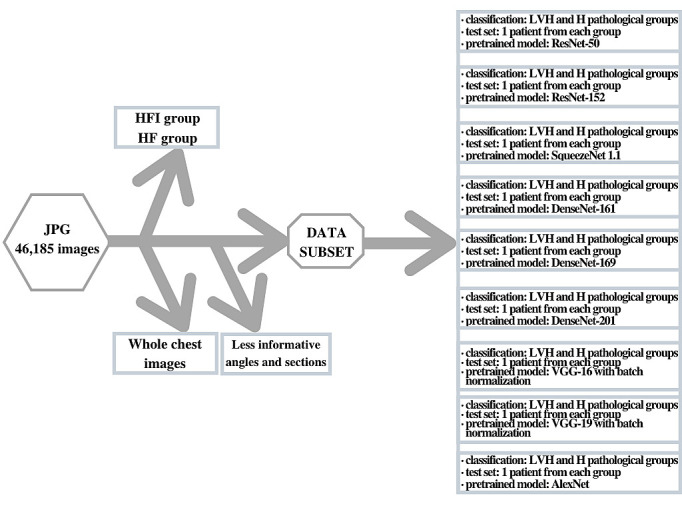
The second set of experiments. Each experiment was conducted with and without data augmentation. HFI – heart failure with infarction; HF –heart failure without infarction; LVH – left ventricle hypertrophy; H – healthy.

An example of images included in one batch in one of the experiments in the first set of experiments is shown in Figure 3. To exclude the possibility that the images were too diverse (showing different angles, sizes, and sections of the heart and the surrounding structures) for neural networks to classify them properly, we created a uniform subset of Sunnybrook Cardiac Data images from two pathological groups: LVH and H (Figure 4). An example of images from the new subset that was included into one batch in one of the experiments is also shown in Figure 3.

### Subset analysis using other pretrained models

To exclude the possibility that the performance in some settings resulted from the performance of a particular pretrained model on MR images, we classified two disease groups (LVH and H) using the created uniform data subset and pretrained models: ResNet-50 (10), ResNet-152 (10), SqueezeNet 1.1 (12), DenseNet-161 (13), DenseNet-169 (13), DenseNet-201 (13), VGG-16 with batch normalization (14), VGG-19 with batch normalization (14), and AlexNet (15). Other experimental settings are shown in Table 1. The test set consisted of MR images of one patient from each group (Figure 4).

### Subset analysis without data augmentation

To exclude the possibility that data augmentation affected the results, we classified two disease groups (LVH and H) with and without data augmentation using the created uniform data subset and pretrained models: ResNet-50 (10), ResNet-152 (10), SqueezeNet 1.1 (12), DenseNet-161 (13), DenseNet-169 (13), DenseNet-201 (13), VGG-16 with batch normalization (14), VGG-19 with batch normalization (14), and AlexNet (15). Other experimental settings are shown in Table 1. The test set consisted of MR images of one patient from each group (Figure 4).

## RESULTS

The error rates were ten times higher in the experimental settings that used MR images of different patients for training and test sets compared with experimental settings that used subsets of MR images of the same patients for training and test sets (Table 2).

**Table 2:** Experimental settings and effects of different test-set designs

Classification	Number of classes	Chest images	Test set	Error rate (%)	Training loss<<Test loss
Disease groups	4	+	10% random images from each class	2.0643	-
Disease groups	4	+	images of one patient from each class	46.7975*	+
Disease groups	4	-	10% random images from each class	1.2866	-
Disease groups	4	-	images of one patient from each class	49.0891*	+
Sex	2	+	10% random images from each class	0.9597	-
Sex	2	+	images of one patient from each class	16.0180*	+
Sex	2	-	10% random images from each class	0.3040	-
Sex	2	-	images of one patient from each class	23.5596*	+
Patients	45	+	10% random images from each class	1.5293	-
Patients	45	-	10% random images from each class	0.3939	-

*We used early stopping in models where the test set consisted of the images of one patient from each class. In the cases where test sets consisted of 10% of random images from each class, both training loss and test loss were decreasing constantly, so there was no need or opportunity for early stopping.

Other pretrained models had a performance comparable with ResNet-50 in the classification task involving two disease groups and using the created uniform data subset where test sets consisted of MR images of one patient from each group (Table 3). Error rates were higher when data augmentation was applied (Table 3).

**Table 3:** Subset analysis using other pretrained models, with and without data augmentation*

Pretrained models	Error rate (%) with data augmentation	Error rate (%) without data augmentation	Training loss<<Test loss
ResNet-50	46.9799	30.2013	**+**
ResNet-152	38.2550	32.2148	**+**
SqueezeNet 1.1	48.9933	25.5034	**+**
DenseNet-161	45.6376	27.5168	**+**
DenseNet-169	32.8859	29.5302	**+**
DenseNet-201	40.2685	33.5570	**+**
VGG-16 with batch normalization	34.8993	32.8859	**+**
VGG-19 with batch normalization	29.5302	14.0940	**+**
AlexNet	53.6913	53.0201	**+**

*Early stopping was applied in all training runs.

## DISCUSSION

In this research, the effects of various test set strategies were determined by measuring the difference in error rates between settings where the test set consisted of random MR images from each class and where images of different patients were used for the training and test sets while keeping all other parameters and settings identical. In the second set of experiments, we excluded some factors other than test set design, which could have contributed to this difference. To the best of our knowledge, no other research has studied the effect of different test-set strategies for medical image classification tasks using machine learning.

We used Sunnybrook Cardiac Data MR images for the classification of four heart disease groups. This database in its raw form might be considered excessively diverse: although all MR images we used show hearts with a particular disease, they are actually sets of many smaller subsets of MR images, each showing a different angle, size, and section of the heart. This could explain why the error rates were so high in the settings where one patient was used as the test set. The error rate would probably have been much lower if we had used MR images of just one section, or if we had classified MR images in more classes representing sections with particular characteristics (pathology or sex). In terms of accuracy, one should also keep in mind that we did not perform any hyperparameter tuning. Creating state-of-the-art models was not a goal of our study and the chosen experimental settings serve to show the importance of choosing the right test set when classifying medical images.

The main reason for conducting all the classifications twice (with and without whole-chest MR images) was to investigate if whole-chest images were misleading the models. At the same time, we validated our results in each experimental setting. When we excluded whole-chest MR images in the settings where training loss was higher than test loss, error rates were lower and error rate measuring was more precise. We can conclude that whole-chest images were indeed misleading the models, which would also be the case with trained cardiologists or radiologists.

In all experimental settings that used different patients in training and test sets, test loss was much greater than training loss, which is a sign of overfitting. This means that the model fits the training data well, but not the test (unseen) data (16). It is learning the noise rather than the characteristics relevant for classification; as a result, the model is not generalizing well (17). In our experiments, this was happening from the first to the last epoch. Since in the first epoch the model was seeing the images for the first time, this phenomenon might be explained by the “too sliced” nature of MR images; even during the first epoch of the training, the model already saw many similar MR images. Due to the high amount of noise and overfitting in such cases (as values of training and test loss show during the training), more training data or training data of higher quality could also help in building better-performing models (16).

Overfitting is caused by a high extent of irrelevant attributes (18). In this case, surrounding tissues are irrelevant and redundant for disease classification. We added patient classifications to test if the model was fitting to the general patient anatomy when random images from each class were used for the test set. This might indeed be the case as the model predicted the patients better (45-class classification) than it predicted the disease (four-class classification).

**Figure 5:**
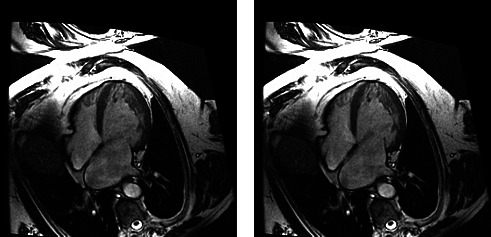
An example of neighboring slices. Image CAP_SCD0001401_MR__hrt_raw_20120813121634880_31 of patient 14 (left) and image CAP_SCD0001401_MR__hrt_raw_20120813121634905_32 of patient 14 (right).

Our results show that error rates are ten times higher when MR images of different patients are used for training and test set than when random subsets of MR images are used. This huge difference is a result of the data leakage effect, which could appear each time when multiple images of the same patients are used in machine learning tasks. Data leakage in machine learning occurs when the data we use to train a model carry the information that we are trying to predict (19). In the case of MR images, this happens if images of the same patients are used for the training and test sets, and images of neighboring slices appear in the training and test set. Since MR images of neighboring slices are very similar (an example is shown in Figure 5), the model behaves in a manner as if it has already seen the answer to the question we are asking; all of this causes overfitting. During this process, the model is learning not only the information needed for accurate prediction but also the noise. This effect is present each time when we use images from the same patients for the training and test sets, but it is more subtle when we use just a few images per patient, and more prominent when we use many images (as in this case). In both cases, the model is learning the general anatomy of the patient rather than characteristics needed for the classification: when it sees the same anatomy (patient) in the test set, it classifies the image properly but based on the wrong characteristics. This in itself does not represent a problem, but it might lead to very misleading conclusions; the model performance is overestimated and the generalization is poor, which leads the model to make wrong predictions for new (unseen) images. This is of particular interest when models are used for medical applications, where they might cause errors leading to patient harm.

**Figure 6:**
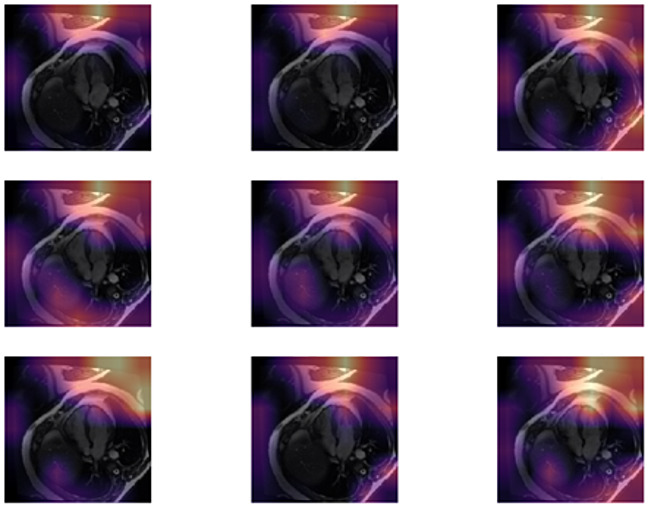
An example of top losses in subset analysis using ResNet-50 for pathological group classification.

To interpret our models’ behavior, we used a technique proposed by Selvaraju et al (20), which highlights the discriminative areas for classification of a given image (the deeper the highlighted color, the more relevant the region is for a particular class prediction). In images shown as top losses in our experiments, the areas that mostly contributed to the decision for the classification were always the structures around the heart, and not the heart itself (Figure 6).

As a PubMed search showed, many machine learning tools for medical image classification are still built in settings that allow the described atypical data leakage. This kind of data leakage might be very discrete but carries serious consequences. In research papers, both test set design strategies are present. Usually, the ones with the test set containing random images from each class have very high accuracy (more than 95%) and a low error rate. On the other hand, those using different patients for training and test sets have much lower accuracy (60%-90%). It is important to be aware of this phenomenon because it might remain unnoticed in less “extreme” data sets and settings.

Augmentation methods do not perform well when images in the training set are similar (21). In our study, in subset analysis, a lower error rate was achieved when data augmentation was not applied. This suggests that data augmentation makes the “too sliced” situation worse; it adds even more similar slices during model training. However, in our study, data augmentation was not decisive for the discrepancy between the error rates observed in experimental settings that used MR images of different patients and experimental settings that used MR images of the same patients for training and test sets.

The study is subject to several limitations. The settings used in this study undoubtedly provoke overfitting in many ways. However, through the use of this method, we were able to show an overemphasized example of the phenomenon that may have remained otherwise unrecognized.

In all experimental settings that used different patients in training and test sets, test loss was much greater than training loss, and the test loss curve was fluctuating (probably due to the nature of the data set), which made error rate measuring less precise.

Future research should propose tools or methods for removing the data leakage effect when deep learning is applied to tasks involving medical images. Since medical image data sets are very small compared with other data sets used in computer vision (21), each image is precious.

Further investigations are also needed to determine if other types of medical data and signals also have the “individual patient’s signature,” and, if so, if other machine learning classification algorithms (random forests, support vectors machines, etc) suffer equally from this phenomenon. More studies are also required to establish the best practices for building and validating machine learning tools in medical image analysis.

In conclusion, when deep learning is applied to MR image classification, training and test sets should be made of different patients’ MR images. Test sets consisting of MR images from patients who have other MR images in the training set yield wrong results: MR images of neighboring slices in the two different sets are so similar that they cause data leakage effect. The models built on training and test sets consisting of random subsets of images of the same patients have optimistic error rates, would not be useful in real clinical situations, and could harm patients. Machine learning tools, therefore, must be validated in rigorous and multicenter settings before they can be clinically used.
